# Oxidative Stress and Uveitis: Molecular Mechanisms and Pathogenetic Role

**DOI:** 10.3390/cimb48090903

**Published:** 2026-09-03

**Authors:** Jiaxin Deng, Yaru Zou, Mingming Yang, Jing Zhang, Wendong Gu, Zizhen Ye, Yuan Zong, Kyoko Ohno-Matsui, Koju Kamoi

**Affiliations:** Department of Ophthalmology & Visual Science, Graduate School of Medical and Dental Sciences, Institute of Science Tokyo, Tokyo 113-8510, Japan; dengjiaxin.med@gmail.com (J.D.); alicezouyaru519@gmail.com (Y.Z.); yangmm-12@outlook.com (M.Y.); zhangj.c@foxmail.com (J.Z.); gwd981107@gmail.com (W.G.); yezizhen518@gmail.com (Z.Y.); zongyuan666@gmail.com (Y.Z.); k.ohno.oph@tmd.ac.jp (K.O.-M.)

**Keywords:** oxidative stress, reactive oxygen species, uveitis, experimental autoimmune uveitis, inflammation, ocular diseases, mitochondrial dysfunction, redox signaling

## Abstract

Uveitis comprises a heterogeneous group of intraocular inflammatory diseases that differ in anatomical location, etiology, clinical course, and immune mechanisms. Despite this diversity, evidence derived predominantly from experimental autoimmune uveitis (EAU) suggests that oxidative stress may act as a context-dependent amplifier linking immune activation, ocular barrier dysfunction, and tissue injury. Excess reactive oxygen species (ROS) disrupt redox homeostasis and activate redox-sensitive inflammatory signaling, thereby amplifying cytokine production, mitochondrial dysfunction, leukocyte recruitment, and retinal damage. This review summarizes current evidence on oxidative stress in uveitis, focusing on major ROS-generating systems, NF-κB-driven inflammation, Nrf2/HO-1-mediated antioxidant responses, MAPK and PI3K-Akt signaling, and immune–metabolic pathways involved in autoimmune ocular inflammation. Cell-specific responses in retinal and immune cells further illustrate how redox imbalance may contribute to local tissue injury and immune-mediated disease progression. The role of oxidative stress in blood–ocular barrier dysfunction, particularly blood–retinal barrier disruption during posterior segment inflammation, is also discussed. Overall, preclinical studies support an active role for oxidative stress in inflammatory amplification and ocular tissue injury, whereas evidence from patients with defined uveitic entities remains limited, heterogeneous, and largely associative. Redox-directed treatments should therefore currently be regarded as investigational adjunctive strategies rather than established therapies for uveitis.

## 1. Introduction

Uveitis encompasses a heterogeneous group of intraocular inflammatory diseases rather than a single clinical entity. The uveitides comprise more than 30 distinct disorders characterized by inflammation within the eye, which may involve the uveal tract and adjacent structures, including the retina, vitreous, and optic nerve [1,2]. Importantly, when inflammation involves the posterior segment, disruption of the blood–retinal barrier (BRB) may occur [3], leading to vascular leakage and facilitating the entry of inflammatory cells and soluble mediators into normally protected retinal compartments [4,5]. Although these disorders are traditionally classified according to the primary anatomical site of inflammation into anterior, intermediate, posterior uveitis, or panuveitis, diseases affecting the same anatomical compartment may differ substantially in etiology, clinical course, prognosis, and underlying immune mechanisms [1,6]. Broadly, uveitis may arise from infectious processes, immune-mediated or autoinflammatory disorders, trauma or other tissue-damaging insults, or remain undifferentiated. Infectious uveitis can be caused by diverse pathogens, including viruses, bacteria, fungi, and parasites. In contrast, non-infectious uveitis is commonly associated with immune dysregulation. Key mechanisms include Th1- and Th17-driven inflammatory responses, insufficient regulatory T-cell control, and immunogenetic susceptibility, such as HLA-associated disease [6,7,8,9].

This etiologic and mechanistic heterogeneity highlights the need to identify convergent molecular pathways that amplify intraocular inflammation and promote tissue injury. Among the molecular pathways that may contribute to this process, oxidative stress has gained increasing attention because reactive oxygen species can enhance inflammatory signaling, disturb blood–ocular barrier homeostasis, and interact with pathogenic immune responses [10,11,12,13,14,15]. Therefore, clarifying the role of oxidative stress in uveitis may provide mechanistic insight across distinct uveitic entities and help explain how diverse triggers converge on ocular inflammatory damage.

Oxidative stress refers to a state of redox imbalance that arises when the production of reactive oxygen species (ROS) exceeds the capacity of antioxidant defense systems. ROS are mainly derived from molecular oxygen and include superoxide anion (O_2_^•−^), hydrogen peroxide (H_2_O_2_), peroxyl radicals (ROO^•^), and the highly reactive hydroxyl radical (^•^OH) [16,17]. By contrast, oxidative molecular damage refers to chemical modifications of cellular lipids, proteins, and nucleic acids caused by excessive or inadequately controlled reactive species. Under physiological conditions, low to moderate levels of ROS contribute to cellular signaling. To prevent excessive ROS accumulation, cells rely on multiple antioxidant defense systems. These include non-enzymatic antioxidants, such as vitamins A, C, and E, glutathione, α-lipoic acid, and carotenoids; trace elements involved in antioxidant activity, such as copper, zinc, manganese, and selenium; and enzymatic scavengers, including superoxide dismutase, catalase, and glutathione peroxidase [18,19]. However, when ROS generation exceeds the capacity of these protective mechanisms, redox homeostasis is disrupted, leading to oxidative molecular damage, including lipid peroxidation, protein oxidation or nitration, and oxidative damage to nuclear or mitochondrial DNA [20]. Persistent oxidative stress can ultimately impair cellular function and tissue integrity across different ocular compartments. In the anterior segment, exposure to ultraviolet radiation, visible light, atmospheric oxygen, and environmental toxins can promote ROS generation in the tear film, cornea, aqueous humor, and lens [21,22,23]. In the posterior segment, high metabolic demand and mitochondrial activity further contribute to ROS production in the retina and choroid [24,25,26]. Thus, both redox imbalance and oxidative molecular damage may occur throughout the eye, providing a biological basis for investigating their roles across different anatomical forms of uveitis.

Importantly, despite the heterogeneity of uveitis in anatomical location, etiology, and clinical presentation, oxidative stress and inflammation may interact through shared molecular pathways that contribute to disease progression and ocular tissue injury. This redox–inflammatory interplay provides a useful framework for understanding how diverse pathogenic triggers may converge on common mechanisms, including mitochondrial dysfunction, blood–ocular barrier disruption, and oxidative stress-associated cell damage.

In this review, we summarize current evidence regarding the role of oxidative stress in the pathogenesis of uveitis, with particular emphasis on redox-sensitive inflammatory signaling, mitochondrial dysfunction, blood–retinal barrier disruption, and oxidative stress-associated cell injury. We further discuss the therapeutic implications of targeting oxidative stress-related pathways in uveitis.

## 2. Sources of Reactive Oxygen Species in Uveitis

Mitochondrial oxidative phosphorylation and the nicotinamide adenine dinucleotide phosphate (NADPH) oxidase (NOX) system are two major endogenous sources of ROS [27].

Mitochondria are considered a major endogenous source of ROS [28]. Under physiological conditions, electrons derived from NADH and FADH_2_ are transferred through complexes I, II, III, and IV of the mitochondrial electron transport chain to reduce oxygen to water, while protons are pumped across the inner mitochondrial membrane to generate the electrochemical gradient required for ATP synthesis [29,30]. During this process, a small proportion of electrons may leak from the electron transport chain and react with molecular oxygen, leading to the formation of superoxide (O_2_^•−^) and other ROS [25].

NOX enzymes are another major cellular source of ROS [31]. They are membrane-associated enzyme complexes that use NADPH as an electron donor to reduce molecular oxygen and generate superoxide. Different NOX isoforms are either co-expressed or preferentially expressed in specific cell types, including NOX2 in phagocytes, NOX1 in colon epithelial cells, NOX4 in vascular cells, and NOX5 in lymphoid tissue cells. NOX expression is mainly regulated at the transcriptional level, although epigenetic regulation has also been described [32]. Upon activation, electrons are transferred to flavin adenine dinucleotide (FAD), then through heme groups, and finally to molecular oxygen, resulting in the production of superoxide anions [31]. In addition to contributing to pathological oxidative stress, ROS generated by NOX enzymes also participate in physiological immune processes, including microbial killing and inflammasome activation [31].

ROS can also be generated as byproducts of various metabolic processes. For example, xanthine oxidase (XO) can produce both superoxide and H_2_O_2_ [33]. Under conditions of endothelial dysfunction, endothelial nitric oxide synthase (eNOS) may become uncoupled and generate superoxide instead of nitric oxide (NO) [33]. Moreover, ROS production is closely related to inflammation, as inflammatory cells can release excessive free radicals, while ROS can further stimulate inflammatory responses [34].

In addition to endogenous ROS-generating systems, exogenous factors may also contribute to oxidative stress. Air pollution, tobacco smoke, and ultraviolet (UV) radiation have been described as environmental contributors to ROS generation [35,36,37,38,39]. UV radiation is especially relevant in the ocular context, as it can affect antioxidant defenses, alter nitric oxide synthase activity, and induce oxidative modifications of DNA and other chromophores, leading to increased ROS production [40,41].

In uveitis, oxidative stress appears to arise from both inflammatory cell-derived ROS and local retinal oxidative responses. In EAU, suppression of the ROS response reduces disease severity, retinal inflammatory mediator expression, and nuclear factor-κB (NF-κB) activation, supporting a pathogenic role for ROS in autoimmune ocular inflammation [14]. Infiltrating phagocytes may contribute to ROS production during this process. However, studies of early EAU also show oxidative and nitrosative damage in photoreceptor mitochondria before extensive macrophage infiltration, suggesting that local retinal oxidative stress may occur early in disease development [42]. In parallel, uveitis is associated with BRB disruption and immune-cell infiltration. Leukocyte adhesion, endothelial adhesion molecule upregulation, barrier breakdown, and leukocyte extravasation have been shown to occur in a coordinated sequence during EAU [43,44]. These mechanisms may be particularly relevant to Behçet’s disease-associated uveitis, in which neutrophil hyperactivation, enhanced oxidative burst, and superoxide/ROS production have been linked to the inflammatory and vascular features of the disease [45,46]. Because the biological interpretation of redox measurements depends on whether they reflect reactive species, downstream molecular damage, or composite redox status, Table 1 summarizes the principal species and markers relevant to uveitis research and highlights the limitations associated with their assessment.

## 3. Redox-Sensitive Molecular Pathways and Immune–Metabolic Axes in Uveitis

**Figure 1:**
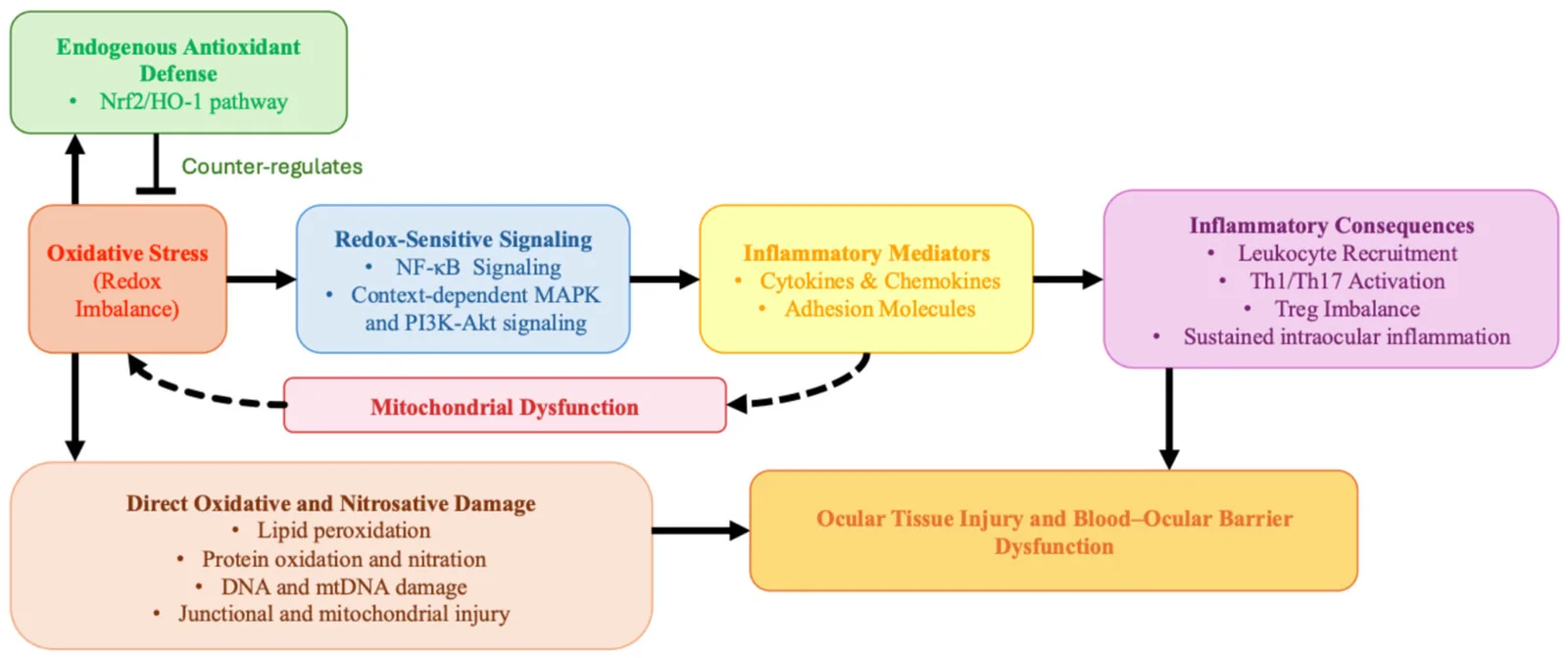
Oxidative stress-driven redox–inflammatory amplification in uveitis. Excess ROS activate redox-sensitive signaling pathways, including NF-κB, MAPK, and PI3K-Akt signaling [14,51,53,54,55,56,57,58,59,60,61]. These pathways induce inflammatory mediators such as cytokines, chemokines, and adhesion molecules, thereby promoting leukocyte recruitment, Th1/Th17 activation and regulatory T-cell (Treg) imbalance, barrier dysfunction, and ocular tissue damage [44,59,60,61,62]. Inflammatory mediators may further contribute to mitochondrial dysfunction, which increases ROS production and reinforces the redox–inflammatory feed-forward loop [52]. The Nrf2/HO-1 pathway functions as an endogenous antioxidant defense system that counter-regulates oxidative stress and may limit inflammatory amplification [63]. Solid arrows indicate the direction of pathway activation or downstream progression. Dashed arrows indicate the mitochondrial dysfunction-associated feed-forward loop that reinforces ROS production and inflammatory signaling. The blunt-ended line indicates the counter-regulatory effect of the Nrf2/HO-1 pathway on oxidative stress.

Oxidative stress and inflammation are tightly interconnected processes that may amplify intraocular inflammation in uveitis (Figure 1). Excessive ROS can activate redox-sensitive signaling pathways, particularly NF-κB, thereby promoting the transcription of pro-inflammatory mediators such as tumor necrosis factor-α (TNF-α), interleukin-1β (IL-1β), and other cytokines [51]. Once established, inflammatory cytokines may further aggravate mitochondrial dysfunction and enhance mitochondrial ROS (mtROS) generation. This creates a feed-forward loop in which ROS-driven inflammatory signaling and cytokine-induced oxidative stress reinforce each other. In parallel, mitochondrial damage may release damage-associated molecular patterns (DAMPs), which can further activate innate immune pathways, including NF-κB signaling and the NLRP3 inflammasome [51,52]. Among these DAMPs, cytosolic mitochondrial DNA (mtDNA) may activate the cyclic GMP–AMP synthase–stimulator of interferon genes (cGAS–STING) pathway and downstream NF-κB signaling [47]. These interactions suggest that redox imbalance in uveitis is not merely a byproduct of inflammation, but may actively participate in sustaining inflammatory signaling, cellular injury, and blood–ocular barrier dysfunction. Therefore, redox-sensitive pathways and inflammasome-related signaling provide important mechanistic links between oxidative stress and the progression of uveitic inflammation [14,42].

### 3.1. NF-κB Signaling: A Redox-Sensitive Amplifier of Ocular Inflammation

The NF-κB signaling pathway represents an important redox-sensitive inflammatory axis in uveitic inflammation. NF-κB activation is not merely a downstream consequence of oxidative stress. ROS can modulate NF-κB signaling at multiple levels, while NF-κB-dependent inflammatory mediators may further promote ROS generation and oxidative tissue injury. Under basal conditions, NF-κB dimers are retained in the cytoplasm by inhibitor of κB (IκB) proteins. Pathological stimuli promote IκB phosphorylation and degradation, allowing NF-κB to translocate into the nucleus and activate the transcription of pro-inflammatory genes, including cytokines, chemokines, adhesion molecules, and enzymes involved in inflammatory tissue damage [64,65].

In EAU, NF-κB appears to function as a redox-sensitive amplifier of ocular inflammation. Experimental evidence shows that suppression of ROS production or antioxidant treatment reduces retinal NF-κB activation, inflammatory mediator expression, and disease severity in EAU [14]. In parallel, inhibition of NF-κB signaling attenuates experimental uveitis and decreases the expression of pro-inflammatory cytokines and chemokines, including TNF-α, IL-1β, IL-6, IL-17, and MCP-1 [53,54,55]. Because EAU is also characterized by adhesion molecule upregulation, leukocyte trafficking, and BRB breakdown, oxidative stress-driven NF-κB activation may provide a mechanistic link between redox imbalance, cytokine production, leukocyte recruitment, and barrier dysfunction [44]. Thus, rather than representing a simple downstream marker of inflammation, NF-κB may help sustain the feed-forward cycle between oxidative stress and intraocular inflammation.

Moreover, in a mouse endotoxin-induced uveitis (EIU) model, resveratrol reduced retinal 8-hydroxy-2′-deoxyguanosine accumulation, NF-κB p65 nuclear translocation, MCP-1 and ICAM-1 expression, and leukocyte adhesion [48]. Similarly, in rat EIU, benfotiamine suppressed oxidative stress-associated NF-κB activation together with aqueous humor leukocyte infiltration, protein accumulation, and inflammatory cytokine and chemokine production [66]. These studies provide additional evidence that oxidative injury and NF-κB activation occur in parallel during acute ocular inflammation. However, because EIU is an acute LPS-driven innate inflammatory model, these findings should not be directly extrapolated to specific forms of human autoimmune uveitis.

Mitochondrial injury and mtDNA release may provide an additional mechanistic link between oxidative stress and NF-κB activation in uveitis. A recent study using peripheral blood samples from patients with uveitis and a rat EAU model reported increased ROS, mitochondrial dysfunction, cytosolic mtDNA accumulation, and activation of the cGAS–STING–NF-κB pathway. In EAU rats, pharmacological reduction in oxidative stress decreased mitochondrial injury, cytosolic mtDNA accumulation, pathway activation, and M1 macrophage polarization. Moreover, cGAS knockdown and STING inhibition attenuated these inflammatory changes, providing additional support for the involvement of an oxidative stress–mtDNA–cGAS–STING–NF-κB axis in experimental uveitis [47].

Overall, available evidence supports NF-κB as a redox-sensitive amplifier of inflammation in experimental uveitis. However, direct evidence for the ROS–NF-κB axis in ocular samples from patients with defined uveitic entities remains limited.

### 3.2. Nrf2/HO-1 Pathway: Endogenous Antioxidant Defense Against Uveitic Inflammation

The Nrf2–Keap1 pathway constitutes a central endogenous defense system against oxidative stress and is important for maintaining redox homeostasis in ocular tissues. Under basal conditions, nuclear factor erythroid 2-related factor 2 (Nrf2) is retained in the cytoplasm by Kelch-like ECH-associated protein 1 (Keap1), which promotes Nrf2 ubiquitination and proteasomal degradation. This maintains low basal transcription of antioxidant genes. In response to oxidative or electrophilic stress, ROS and electrophilic intermediates modify critical cysteine residues on Keap1, disrupting Keap1-mediated repression of Nrf2. Stabilized Nrf2 then translocates into the nucleus, heterodimerizes with small Maf proteins, and binds antioxidant response elements (AREs) in the promoters of cytoprotective genes. Through this mechanism, Nrf2 induces the coordinated expression of antioxidant and detoxifying enzymes, including heme oxygenase-1 (HO-1), NAD(P)H quinone oxidoreductase-1 (NQO1), glutamate-cysteine ligase catalytic subunit (GCLC), and superoxide dismutases, thereby enhancing cellular resistance to oxidative injury [63,67].

Among these downstream targets, HO-1 is particularly relevant to inflammatory ocular diseases because it represents an inducible stress-response enzyme that couples antioxidant defense with anti-inflammatory and cytoprotective effects [68,69]. By degrading pro-oxidant heme and generating biologically active products such as biliverdin, bilirubin, and carbon monoxide, HO-1 can reduce oxidative burden and modulate inflammatory signaling. In RPE cells, HO-1 induction has also been associated with protection against oxidative stress-induced injury [69,70,71,72]. Protective effects of Nrf2 activation have also been reported in chronic ocular diseases such as diabetic retinopathy, glaucoma, and age-related macular degeneration; however, these findings provide only indirect mechanistic context and should not be extrapolated directly to uveitis [67].

In the context of uveitis, these observations support the view that Nrf2/HO-1 functions as an adaptive antioxidant pathway that may counterbalance ROS-driven inflammatory injury. Experimental studies further suggest that pharmacological activation of the Nrf2/HO-1 axis can attenuate ocular inflammation and oxidative stress in uveitis models [73]. In EAU, Nrf2 deficiency exacerbates retinal inflammation, whereas Nrf2 activation reduces clinical and pathological disease severity [74]. Similarly, modulation of HO-1 activity influences EAU severity, with HO-1 induction ameliorating inflammation. Moreover, reduced expression of Nrf2/HO-1 pathway components in EAU suggests that endogenous antioxidant defenses may be insufficient or dysregulated during uveitic inflammation. Therefore, modulation of the Nrf2/HO-1 pathway may represent a promising therapeutic strategy, although its efficacy is likely to vary according to disease stage, inflammatory context, and the mode of pathway activation [73,74].

### 3.3. MAPK and PI3K-Akt Signaling: Context-Dependent Redox-Sensitive Regulators of Ocular Inflammation and Immune Remodeling

Mitogen-activated protein kinase (MAPK) and phosphoinositide 3-kinase-Akt (PI3K-Akt) signaling pathways can be viewed as context-dependent redox-sensitive kinase networks that translate oxidative stress into cellular survival, inflammatory activation, immune remodeling, and tissue injury in ocular diseases. In contrast to NF-κB, which mainly amplifies inflammatory gene transcription, and Nrf2/HO-1, which mainly mediates antioxidant and cytoprotective adaptation, MAPK and PI3K-Akt signaling determine how ocular and immune cells respond to stress. These pathways do not have uniformly protective or pathogenic effects. Transient activation may support survival, repair, and metabolic adaptation, whereas sustained activation in a redox–inflammatory environment can promote apoptosis, cytokine production, barrier disruption, angiogenesis, and pathogenic T-cell responses. Therefore, these kinase pathways provide an important mechanistic layer between oxidative stress and chronic inflammatory remodeling in uveitis and other ocular diseases [75].

The MAPK family includes extracellular signal-regulated kinase (ERK), c-Jun N-terminal kinase (JNK), and p38 MAPK. These kinases respond to oxidative and inflammatory stimuli but mediate different cellular outcomes depending on the duration and cellular context of activation. ERK activation is often associated with survival and adaptive repair, whereas prolonged activation of stress-responsive JNK and p38 MAPK can promote mitochondrial dysfunction, apoptosis, inflammatory gene expression, and tissue injury [75]. In chronic retinal diseases such as DR and AMD, persistent oxidative stress has been linked to neurovascular injury, RPE dysfunction, inflammatory amplification, and barrier impairment, although the specific involvement of MAPK signaling may vary by disease context [25,26,75]. However, these findings provide only indirect mechanistic context for uveitis.

In uveitis, MAPK signaling appears to contribute to the amplification of autoimmune and inflammatory responses. In EAU, increased phosphorylation of p38 MAPK, ERK1/2, and JNK has been observed in inflamed retinal tissue, and suppression of these signaling pathways is associated with reduced ocular inflammation [58]. Experimental studies also suggest that p38 MAPK participates in uveitogenic T-cell responses. Inhibition of p38 MAPK reduces interphotoreceptor retinoid-binding protein (IRBP)-induced interferon-γ (IFN-γ) and interleukin-17 (IL-17) production, and p38-MAPK-related modulation has been linked to reduced CD4^+^ T-cell activation, altered effector T-cell differentiation, and attenuation of EAU severity [59,60]. These findings indicate that MAPK signaling may connect oxidative stress and inflammatory stimuli with cytokine production, Th1/Th17 responses, and retinal inflammatory injury in autoimmune uveitis.

PI3K-Akt signaling represents a parallel redox-regulated kinase pathway that integrates survival, metabolism, and immune-cell function. In retinal cells, Akt activation can promote cell survival, preserve mitochondrial integrity, and counteract oxidative injury. However, persistent activation under pathological conditions may support maladaptive remodeling, including inflammatory responses and, in vascular retinal diseases, angiogenic signaling through hypoxia-inducible factor-1α (HIF-1α) and vascular endothelial growth factor (VEGF) [56,57]. In uveitis, the most relevant role of PI3K-Akt signaling may not be angiogenesis alone, but immune–metabolic regulation. Recent EAU evidence suggests that PI3K-Akt-related metabolic pathways can influence CD4^+^ T-cell glycolysis, Th17/Treg balance, granulocyte-macrophage colony-stimulating factor (GM-CSF) production, and inflammatory disease severity [61]. This places PI3K-Akt signaling at the intersection of oxidative stress, metabolic reprogramming, and adaptive immune dysregulation.

In uveitis, available evidence supports a role for these pathways in cytokine production, uveitogenic T-cell responses, and inflammatory tissue injury, although their specific contribution may vary by cell type, disease stage, and redox context. Collectively, MAPK and PI3K-Akt should be viewed not as uniformly pathogenic pathways, but as redox-sensitive molecular decision points that may shift ocular inflammation from reversible stress responses toward persistent immune-mediated damage.

## 4. Cell-Specific Effects of Oxidative Stress

**Figure 2:**
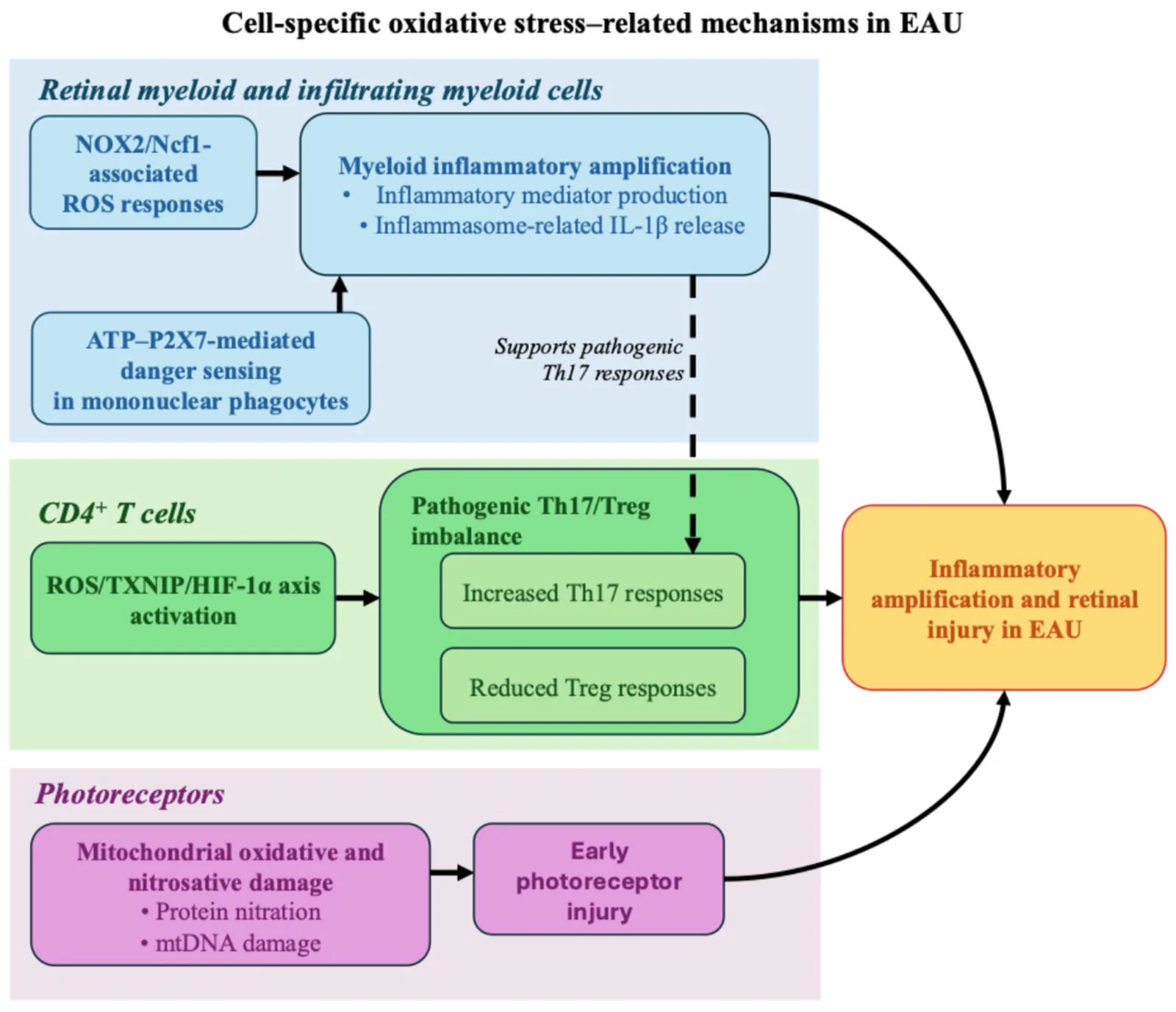
Proposed cell-specific oxidative stress-related mechanisms in experimental autoimmune uveitis. This conceptual model summarizes evidence derived predominantly from experimental autoimmune uveitis (EAU) and related ex vivo studies. In the myeloid compartment, NOX2/Ncf1-associated ROS responses and ATP–P2X7-mediated danger sensing in mononuclear phagocytes contribute to inflammatory mediator production and inflammasome-related IL-1β release, thereby amplifying retinal inflammation [14,76]. Myeloid inflammatory responses may also support pathogenic Th17 responses. In CD4^+^ T cells, activation of the ROS/TXNIP/HIF-1α axis favors a pathogenic Th17/Treg imbalance [62]. In photoreceptors, early mitochondrial oxidative and nitrosative damage, including protein nitration and mtDNA damage, may contribute to photoreceptor injury before extensive inflammatory cell infiltration [15,42]. Together, these cell-specific mechanisms may promote inflammatory amplification and retinal injury in EAU. Solid black arrows indicate mechanistic progression within individual cellular compartments or contributions to the shared EAU outcome. The dashed black arrow indicates a proposed cross-cell interaction that is indirectly supported by the available evidence. Background colors distinguish cellular compartments and do not indicate differences in evidence strength.

The major cell-specific effects of oxidative stress in uveitis are summarized in Figure 2, with emphasis on photoreceptors, CD4^+^ T cells, and retinal myeloid cells.

In EAU, photoreceptors appear to be affected by oxidative stress at an early stage of disease and are not merely passive targets of subsequent inflammatory injury. Mitochondrial oxidative damage, including mitochondrial protein nitration and mitochondrial DNA damage, has been detected before prominent inflammatory cell infiltration. These findings suggest that photoreceptor mitochondria may represent an early site of redox imbalance during autoimmune retinal inflammation [42]. This oxidative injury is selectively observed in the photoreceptor inner segments, where mitochondria are highly enriched, and has been associated with increased inducible nitric oxide synthase (iNOS) expression in photoreceptor mitochondria as well as retinal cytokine production triggered by a small number of antigen-specific infiltrating T cells [42]. Nguyen et al. indicated that even limited immune-cell entry into the retina may be sufficient to initiate local inflammatory signaling and mitochondrial oxidative stress in photoreceptors. In this context, innate immune activation may also contribute to early retinal injury [15]. TLR4-mediated innate immune responses may promote inflammatory cytokine production, leading to oxidative DNA damage and mitochondrial dysfunction before extensive inflammatory cell infiltration occurs [15]. Subsequently, adaptive immune responses may further amplify cytokine expression and mitochondrial oxidative stress, thereby sustaining photoreceptor injury during EAU.

Additionally, in adaptive immune cells, oxidative stress may influence the differentiation and pathogenicity of uveitogenic T cells. Evidence from EAU indicates that melatonin can attenuate ocular inflammation by modulating CD4^+^ T-cell responses through the ROS/TXNIP/HIF-1α axis. In this model, melatonin did not simply act as a general antioxidant. Rather, it reduced intracellular ROS accumulation in CD4^+^ T cells and suppressed TXNIP/HIF-1α signaling. It also limited the differentiation and inflammatory activity of Th17 cells while promoting Treg responses. This redox-dependent regulation of the Th17/Treg balance suggests that oxidative stress is directly involved in controlling pathogenic adaptive immune responses during autoimmune uveitis [62]. Therefore, the ROS/TXNIP/HIF-1α pathway provides a cell-specific mechanism through which oxidative stress can promote ocular autoimmunity, and its inhibition may represent a strategy for restraining uveitogenic T-cell responses and alleviating retinal inflammation.

Moreover, in retinal myeloid cells, oxidative stress may further sustain autoimmune uveitis by interacting with innate danger-sensing pathways that link local inflammation to adaptive immune activation. Evidence from EAU supports a functional role for ROS in this process. Suppression of the reactive oxygen response, either through Ncf1 deficiency or antioxidant treatment with N-acetylcysteine, attenuates EAU severity and reduces retinal oxidative injury, inflammatory mediator production, and NF-κB activation. These findings indicate that ROS are not merely secondary by-products of retinal inflammation, but active contributors to the propagation of autoimmune ocular inflammation. Because Ncf1 is an essential component of the NOX2 complex, which is closely associated with phagocyte oxidative responses, this evidence also suggests that myeloid-cell-associated ROS may participate in inflammatory amplification within the retina [14].

This redox-sensitive inflammatory environment can be further linked to mononuclear phagocyte activation through P2X7-mediated danger sensing. P2X7 is an ATP-gated receptor expressed by mononuclear phagocytes and T cells [77]. As extracellular ATP can be released from stressed or damaged cells, P2X7 functions as an important danger-sensing receptor in inflammatory tissues. In mononuclear phagocytes, P2X7 activation links tissue stress to NLRP3 inflammasome activation, maturation of IL-1β and IL-18, ADAM17-mediated TNF-α shedding, and the production of ROS and IL-6 [77,78]. P2X7 has also been implicated in T-cell activation and Th1/Th17 polarization, although whether these effects are mediated directly through T cells or indirectly through neighboring innate immune cells may depend on the cellular context [77]. In EAU, recent cell-specific evidence indicates that P2X7 acts predominantly through mononuclear phagocytes rather than CD4^+^ T cells [76]. P2X7 deficiency in mononuclear phagocytes reduces disease severity and retinal immune-cell infiltration [76]. At the mechanistic level, loss of P2X7 in monocyte-derived macrophages downregulates inflammasome-, phagocytosis-, and complement-related pathways, impairs IL-1β release, and is associated with reduced pathogenic Th17 responses in the retina. In microglia, P2X7 deficiency particularly affects an IFN-responsive subset characteristic of EAU [76]. These findings suggest that infiltrating macrophages and resident microglia may provide a cell-specific platform where ROS-dependent inflammatory signaling and ATP/P2X7-mediated danger sensing converge, linking oxidative stress, inflammasome activation, complement regulation, and Th17-mediated autoimmune retinal inflammation.

## 5. Oxidative Stress and Ocular Barrier Dysfunction

Ocular barrier dysfunction in uveitis may involve both blood–aqueous and blood–retinal barriers. In anterior uveitis, disruption of the normally controlled blood–aqueous interface is reflected clinically by inflammatory cells and protein flare in the anterior chamber [1,2]. By contrast, posterior or panuveitic inflammation is more closely associated with BRB dysfunction [3,4,5,44,79,80].

The anterior segment is also susceptible to oxidative stress because of its exposure to light, atmospheric oxygen, and environmental stimuli. At the same time, the aqueous humor and anterior uveal tissues contain antioxidant defense systems that help maintain local redox homeostasis [34]. When ROS production exceeds local antioxidant capacity, oxidative stress may contribute to inflammatory amplification in the anterior segment [34,81]. In experimental anterior uveitis models, anterior chamber cell infiltration and increased aqueous humor protein are commonly used as indicators of blood–aqueous barrier disruption and inflammatory exudation. Consistent with this concept, antioxidant interventions in experimental anterior uveitis have been reported to reduce aqueous humor cellular infiltration, total protein levels, and inflammatory mediators such as PGE_2_, NO, TNF-α, IL-6, CCL2, and MIP-2. These findings suggest that oxidative stress may participate in anterior segment barrier dysfunction and inflammatory exudation, although the direct evidence is still more limited than that for BRB disruption in posterior segment diseases [81].

The BRB is a highly selective barrier system that separates the neural retina from the systemic circulation and maintains retinal homeostasis by regulating the exchange of nutrients, metabolites, ions, and waste products. It consists of the inner BRB, formed primarily by retinal vascular endothelial cells and their tight junctions, and the outer BRB, formed by tight junctions between RPE cells [79,80]. In uveitis, especially in EAU and non-infectious posterior uveitis, BRB dysfunction is associated with retinal endothelial activation, increased expression of adhesion molecules, downregulation or redistribution of tight junction proteins, vascular leakage, and leukocyte recruitment [44]. Therefore, BRB breakdown should be viewed not simply as a consequence of inflammation but also as a mechanism that amplifies intraocular inflammatory responses.

Oxidative stress may contribute to BRB disruption through several complementary mechanisms. First, excessive ROS can directly impair junctional integrity in retinal barrier cells by altering tight junction proteins and cytoskeletal organization. Intercellular junctional proteins, including occludin, claudins, and zonula occludens-1 (ZO-1), are essential for maintaining BRB integrity, and their loss, phosphorylation, or abnormal localization is closely associated with increased barrier permeability [82,83]. Second, oxidative stress can promote inflammatory signaling pathways such as NF-κB, thereby enhancing the production of cytokines, chemokines, and adhesion molecules that favor endothelial activation and leukocyte trafficking [14]. Third, ROS-mediated barrier injury may interact with VEGF-dependent permeability pathways in retinal cells. However, direct evidence for a complete ROS–VEGF–BRB axis in uveitis remains limited, because most supporting evidence derives from diabetic retinopathy or in vitro retinal barrier models [25,84]. Therefore, this pathway should be regarded as mechanistically plausible rather than established in uveitis. The major oxidative stress-related pathways and cellular mechanisms discussed above are summarized in Table 2, including their main cell types, mechanistic roles, supporting evidence, and potential therapeutic relevance.

## 6. Disease Relevance and Therapeutic Translation

The translational relevance of oxidative stress varies considerably among uveitic entities. Most evidence supporting an active pathogenic role for ROS has been obtained from EAU, EIU, and associated in vitro or ex vivo mechanistic studies [14,47,48,62,66,76]. Human studies are fewer and differ substantially in disease classification, biological samples, measured biomarkers, disease activity, and treatment exposure. Accordingly, oxidative stress cannot yet be regarded as a universally established initiating mechanism of uveitis. Current evidence more strongly supports its role as a context-dependent contributor that may amplify immune activation, blood–ocular barrier dysfunction, and ocular tissue injury. Given the limited and heterogeneous nature of patient-based evidence, Table 3 provides an overview of the available human studies across different uveitic entities and highlights the main limitations affecting their disease-specific interpretation.

### 6.1. Human Evidence and Disease-Specific Relevance

Human evidence remains uneven across biological sample types. In the literature identified for this review, available studies mainly involved serum, peripheral blood cells, and aqueous humor. We did not identify robust studies measuring specific ROS or validated oxidative molecular damage products in vitreous, tears, or ocular tissue samples from well-characterized human uveitis cohorts. Therefore, the absence of evidence from these compartments should not be interpreted as evidence that redox abnormalities are absent, but rather as an important gap in the current literature.

Evidence for redox abnormalities in defined infectious uveitic entities is particularly limited. Moreover, because ROS participate in physiological antimicrobial host defense, findings obtained from EAU and other autoimmune inflammation models should not be directly extrapolated to infectious uveitis. The biological consequences of suppressing ROS may differ substantially between immune-mediated and pathogen-driven ocular inflammation.

#### 6.1.1. Behçet’s Uveitis

Behçet’s uveitis represents one of the more biologically plausible settings in which oxidative stress may be linked to a disease-characteristic inflammatory phenotype. Neutrophil hyperactivation is a recognized feature of Behçet’s disease, and activated neutrophils can generate superoxide and other reactive species through an enhanced oxidative burst. These responses may interact with endothelial activation, thrombosis, and vascular inflammation, which are particularly relevant to the recurrent occlusive retinal vasculitis observed in Behçet’s uveitis [45,46].

Recent proteomic analysis of neutrophils obtained from patients with Behçet’s uveitis has provided disease-specific human evidence that neutrophil functional abnormalities are associated with ocular involvement [45]. Nevertheless, this evidence should be interpreted carefully. The study assessed circulating neutrophils rather than aqueous humor, vitreous, retinal vessels, or ocular tissue, and therefore does not directly demonstrate increased ROS production within the inflamed eye. Similarly, much of the literature relating oxidative burst to vascular injury concerns systemic Behçet’s syndrome rather than isolated ocular disease [46].

Thus, the available evidence supports oxidative and neutrophil-associated mechanisms as potentially relevant to the inflammatory and vascular phenotype of Behçet’s uveitis, but it does not yet establish intraocular oxidative stress as an independent determinant of ocular activity or prognosis. Studies correlating neutrophil oxidative activity with retinal vasculitis, vascular occlusion, imaging findings, and ocular-fluid oxidative markers would be required to strengthen this disease-specific relationship.

#### 6.1.2. Acute Anterior Uveitis and HLA-B27-Associated Uveitis

Human evidence for oxidative abnormalities in acute anterior uveitis remains limited. Türk et al. prospectively compared 45 patients with a first episode of acute anterior uveitis with 43 healthy controls. Serum total oxidant capacity, oxidative stress index, and malondialdehyde levels were significantly higher in the acute anterior uveitis group, whereas total antioxidant capacity did not differ between groups [49]. These findings support the presence of systemic oxidant–antioxidant imbalance and increased lipid peroxidation during active acute anterior uveitis.

The interpretation of these findings is constrained by both the biological sample and the clinical classification. All redox measurements were obtained from serum rather than aqueous humor or ocular tissue, and the underlying causes of acute anterior uveitis were not identified. The observed abnormalities may therefore reflect systemic responses accompanying active ocular inflammation rather than redox changes originating within the anterior chamber. In addition, because only patients experiencing their first episode were included, the study does not clarify whether these abnormalities persist during remission or are associated with recurrent disease [49].

These findings should not be directly extrapolated to HLA-B27-associated acute anterior uveitis. HLA-B27-associated disease is a clinically distinct form of acute anterior uveitis characterized by acute, usually unilateral and alternating non-granulomatous inflammation, frequent recurrences, and marked cellular and protein extravasation into the anterior chamber [9]. However, the cohort reported by Türk et al. was not stratified according to HLA-B27 status, and no comparison of oxidative biomarkers between HLA-B27-positive and HLA-B27-negative acute anterior uveitis was performed. Current evidence therefore supports an association between acute anterior uveitis and altered systemic redox homeostasis, but a disease-specific role for oxidative stress in HLA-B27-associated uveitis remains unproven.

#### 6.1.3. Vogt–Koyanagi–Harada Disease

Direct evidence linking ROS or oxidative molecular damage to Vogt–Koyanagi–Harada (VKH) disease remains sparse. Commodaro et al. showed that p38α MAPK regulates IL-17 production in cells obtained from patients with VKH disease and in EAU [60]. This study provides human evidence connecting a stress-responsive kinase pathway with a disease-relevant Th17 response.

However, p38 MAPK can be activated by numerous inflammatory and cellular stress signals, and its activation does not itself demonstrate increased ROS production or oxidative damage. The available findings should therefore be described as evidence for a potentially redox-sensitive inflammatory pathway, rather than proof of a disease-specific ROS–p38–IL-17 axis. At present, direct measurements of oxidative markers in aqueous humor, cerebrospinal fluid, or ocular tissues from well-characterized patients with active VKH disease remain insufficient.

#### 6.1.4. Evidence from Heterogeneous Uveitis Cohorts

Human ocular-fluid studies provide more direct access to the intraocular environment, although their disease-specific interpretation remains difficult. Yoshida et al. measured pigment epithelium-derived factor (PEDF) and total antioxidant capacity in aqueous humor samples from 34 patients with active uveitis and nine patients with proliferative diabetic retinopathy. The uveitis cohort was clinically heterogeneous and included 13 patients with infectious uveitis and 21 with non-infectious uveitis [50].

Aqueous humor PEDF and total antioxidant capacity were higher in the uveitis group than in the proliferative diabetic retinopathy group, and PEDF was positively correlated with total antioxidant capacity across the studied samples [50]. These findings suggest that PEDF may contribute to endogenous antioxidant capacity within the human eye. In active uveitis, increased PEDF may represent a compensatory response to inflammation or oxidative stress rather than evidence that antioxidant defense is deficient.

Several limitations prevent stronger conclusions. The study did not include a healthy ocular control group, and proliferative diabetic retinopathy itself is associated with profound oxidative and vascular abnormalities. Total antioxidant capacity is also a composite measure that does not identify the dominant reactive species, cellular source, or presence of oxidative molecular damage. Moreover, infectious and non-infectious uveitis were analyzed together without disease-specific subgroup results. The study therefore supports an association between PEDF and intraocular antioxidant capacity but does not establish a common oxidative mechanism across different uveitic entities.

### 6.2. Therapeutic Strategies Targeting Redox–Inflammatory Pathways

Preclinical redox-directed strategies can be broadly divided into three categories: reducing pathological ROS accumulation, enhancing endogenous antioxidant defenses, and modulating downstream redox-sensitive inflammatory pathways.

Direct antioxidant or ROS-modulating interventions have attenuated experimental uveitis through distinct mechanisms. N-acetylcysteine reduced retinal oxidative stress, NF-κB activation, inflammatory mediator expression, and disease severity in EAU [14]. Tempol limited mitochondrial oxidative injury, cytosolic mtDNA accumulation, cGAS–STING–NF-κB activation, and pro-inflammatory macrophage polarization [47], whereas melatonin reduced ROS accumulation in CD4^+^ T cells and modulated the ROS/TXNIP/HIF-1α axis, thereby restoring the Th17/Treg balance [62]. Resveratrol, benfotiamine, lutein, and astaxanthin have also reduced inflammatory responses in EIU [48,66,81,85]. However, these compounds can additionally affect NF-κB signaling, cytokine production, and leukocyte recruitment; their protective effects therefore cannot be attributed exclusively to direct ROS scavenging. Moreover, EIU is an acute LPS-driven model, and efficacy in this setting does not establish therapeutic efficacy in human uveitis.

Enhancement of endogenous antioxidant defenses represents a mechanistically distinct approach. Hyaluronic acid–curcumin nanoparticles attenuated EAU through activation of the Keap1/Nrf2/HO-1 pathway [73], whereas genetic ablation of Nrf2 exacerbated retinal inflammation and disease severity [74]. Together, these findings support a protective role for Nrf2-mediated antioxidant responses, although this strategy has not yet been validated clinically.

Pathway-directed interventions affecting NF-κB, MAPK, PI3K–Akt, cGAS–STING, and P2X7-associated signaling have also reduced inflammatory responses in experimental uveitis [47,53,54,55,58,59,60,61,76]. These approaches may offer greater mechanistic specificity than general antioxidant supplementation, but the targeted pathways are not exclusively regulated by ROS, and their relevance to defined human uveitic entities remains to be established.

### 6.3. Clinical Evidence for Antioxidant Interventions

Clinical evidence for antioxidant intervention in uveitis remains sparse. Van Rooij et al. enrolled 145 patients with acute anterior uveitis in a randomized, double-masked, placebo-controlled trial. Patients received oral vitamin C and vitamin E or matched placebo for 30 days in addition to conventional topical prednisolone and mydriatic treatment. Laser flare and cell measurements were used as objective indicators of anterior chamber inflammation [86].

Vitamin supplementation did not significantly alter the longitudinal course of laser flare, laser cell counts, semiquantitative clinical inflammatory variables, or topical corticosteroid use [86]. These results indicate that adjunctive oral vitamins C and E did not accelerate the resolution of established anterior chamber inflammation. Because all patients received effective topical corticosteroid therapy, however, a modest additional effect may have been difficult to detect.

At 56 days, visual acuity was significantly better in the vitamin group in a separate single-time-point analysis, whereas the overall repeated-measures analysis did not show a significant treatment-related difference in visual acuity over time [86]. This finding should therefore be interpreted as an exploratory functional signal rather than definitive evidence of efficacy. The trial also included both HLA-B27-positive and HLA-B27-negative patients, but it was not designed to establish HLA-B27-specific antioxidant effects and did not incorporate oxidative biomarkers or measurements of ocular target engagement.

### 6.4. Why Have Antioxidant Therapies Shown Variable Clinical Success?

Clinical evidence remains too limited to establish a consistent pattern of antioxidant efficacy in uveitis. Nevertheless, the discrepancy between the generally favorable findings in experimental models and the limited clinical benefit observed to date may be explained by several factors.

One important factor is the difference in disease stage and therapeutic context. Experimental models allow redox-directed interventions to be evaluated at tightly controlled stages of inflammation. By contrast, patients in the trial by van Rooij et al. had already developed clinically apparent acute anterior uveitis and received topical corticosteroids and mydriatic treatment in addition to vitamins C and E [86]. The authors noted that potent corticosteroid therapy may have reduced the ability to detect a modest additional effect of antioxidant supplementation. Once leukocyte infiltration, blood–aqueous barrier disruption, and inflammatory signaling are established, non-specific antioxidants may also be insufficient to reverse the inflammatory process.

Systemic exposure does not necessarily indicate ocular target engagement. Türk et al. identified systemic redox abnormalities in serum [49], whereas van Rooij et al. measured serum vitamin E concentrations but did not assess aqueous humor ROS, oxidative molecular damage, or antioxidant capacity before and after treatment [86]. It therefore remains unclear whether oral supplementation altered the relevant intraocular redox state. Endpoint selection further complicates interpretation: objective measures of anterior chamber inflammation were not improved, whereas the only positive functional signal occurred in visual acuity at the final follow-up point. Visual acuity, however, is influenced by multiple ocular factors and cannot serve as a direct marker of oxidative stress.

Disease heterogeneity and intervention specificity are also likely to influence treatment responses. The acute anterior uveitis cohorts studied by Türk et al. and van Rooij et al. were not restricted to a single etiology, while the Yoshida cohort combined infectious and non-infectious uveitis [49,50,86]. Different uveitic entities may involve different cellular sources, anatomical compartments, and reactive species. Moreover, the association between aqueous humor PEDF and total antioxidant capacity may reflect endogenous counter-regulation rather than a simple antioxidant deficiency requiring replacement [50]. Broad vitamin supplementation may therefore not reproduce the effects of experimental compounds that simultaneously modulate ROS, NF-κB signaling, cytokine production, or other defined pathways. Because ROS also participate in physiological signaling and antimicrobial defense [19,31], selective targeting of pathological ROS sources or redox–inflammatory pathways may ultimately be more appropriate than indiscriminate antioxidant supplementation.

### 6.5. Causal Interpretation and Priorities for Clinical Translation

Genetic or pharmacological suppression of NOX2/Ncf1-associated ROS responses reduced EAU scores and retinal inflammatory mediators [14]. Tempol limited mitochondrial injury, inflammatory macrophage polarization, and ocular tissue damage [47], whereas Nrf2-directed interventions improved clinical and histopathological outcomes in EAU [73,74]. These findings indicate that redox pathways can actively modify experimental disease expression rather than merely reflect tissue injury. However, they do not establish ROS as the initiating trigger of uveitis, because the interventions studied also influence downstream inflammatory signaling, immune-cell responses, and cytoprotective pathways.

Evidence from human uveitis remains primarily associative. Increased serum total oxidant capacity, oxidative stress index, and malondialdehyde in acute anterior uveitis support the presence of systemic redox imbalance and lipid peroxidation during active disease [49]. The association between aqueous humor PEDF and total antioxidant capacity is compatible with intraocular antioxidant regulation but does not directly demonstrate ROS generation or oxidative molecular damage [50]. Moreover, adjunctive vitamins C and E did not suppress objective anterior chamber inflammation in the only randomized clinical trial identified [86]. Taken together, these findings neither confirm nor exclude a causal role for oxidative stress in human uveitis. Redox abnormalities may arise downstream of inflammation and subsequently participate in feed-forward amplification, whereas failure of non-specific vitamin supplementation does not test the therapeutic value of targeting a defined pathological ROS source.

The key translational question is therefore not simply whether oxidative stress accompanies uveitis, but which redox pathway is pathogenic, in which uveitic entity and ocular compartment, and at what stage of disease. Longitudinal studies in clinically well-defined cohorts are needed to determine whether redox abnormalities precede inflammation, track with disease activity, or persist after clinical remission. Where clinically feasible, paired ocular-fluid and peripheral blood sampling could help distinguish intraocular from systemic processes. Candidate biomarkers should separately assess specific ROS-generating pathways, oxidative molecular damage, and compensatory antioxidant responses using analytically validated methods.

Clinical translation should subsequently focus on mechanism-based rather than indiscriminate antioxidant therapy. Early-phase trials should enroll biologically and clinically defined uveitic subgroups, incorporate pharmacodynamic evidence of ocular target engagement, and use prespecified, disease-appropriate inflammatory and visual outcomes. Until such evidence is available, redox-directed agents should be regarded as investigational adjuncts rather than established stand-alone therapies.

## 7. Method of Literature Search

A literature search was conducted to identify studies published in English between January 1980 and May 2026. PubMed and Google Scholar were searched using combinations of terms related to uveitis and oxidative stress, including “uveitis,” “ocular inflammation,” “intraocular inflammation,” “experimental autoimmune uveitis,” “EAU,” “oxidative stress,” “reactive oxygen species,” “ROS,” “redox,” “antioxidant,” “reactive species,” “mitochondrial dysfunction,” “NADPH oxidase,” “lipid peroxidation,” and “oxidative damage.” Additional pathway- and cell-specific terms, including “NF-κB,” “Nrf2,” “HO-1,” “MAPK,” “PI3K-Akt,” “inflammasome,” “photoreceptor,” “retinal pigment epithelium,” “T cell,” “macrophage,” “microglia,” “blood–aqueous barrier,” and “blood–retinal barrier,” were used when appropriate. Human studies, animal models of uveitis, and relevant in vitro studies were included. Studies of other ocular diseases, including diabetic retinopathy, age-related macular degeneration, and glaucoma, were considered only when they provided mechanistic context for pathways for which direct evidence in uveitis was limited; these studies were clearly identified as indirect evidence. Reference lists of relevant articles and reviews were also manually screened to identify additional publications. Non-English-language articles, conference abstracts without sufficient methodological information, and studies without direct relevance to oxidative stress or ocular inflammation were excluded.

## 8. Conclusions

Oxidative stress has emerged as a mechanistically relevant contributor to immune activation, cellular injury, and ocular barrier dysfunction in experimental uveitis models. Although uveitis encompasses diseases with diverse anatomical locations and etiologies, redox imbalance may serve as a convergent but context-dependent pathway through which diverse inflammatory triggers promote tissue damage. Evidence from EAU suggests that ROS are not merely byproducts of inflammation. Instead, they can actively amplify ocular inflammation by activating redox-sensitive pathways such as NF-κB, MAPK, PI3K-Akt, and inflammasome-related signaling. In contrast, antioxidant responses such as the Nrf2/HO-1 pathway may act as endogenous protective mechanisms, although they may be insufficient during sustained inflammation. However, these experimental findings do not establish oxidative stress as a universal initiating mechanism across human uveitic entities.

The effects of oxidative stress in uveitis are cell- and context-dependent. In retinal cells, especially photoreceptors, mitochondrial oxidative damage may contribute to early tissue injury. In immune cells, ROS-related pathways can regulate pathogenic T-cell responses and mononuclear phagocyte activation. Experimental evidence further suggests that oxidative stress may also promote blood–ocular barrier dysfunction by impairing junctional integrity, enhancing inflammatory signaling, and facilitating leukocyte recruitment. These observations support a model in which oxidative stress sustains redox–inflammatory feedback and promotes uveitic tissue damage in experimental settings. In human uveitis, however, the available evidence remains limited, heterogeneous, and primarily associative, and causal relationships have not yet been established. Further studies should determine how specific ROS sources, cell types, ocular compartments, and disease stages shape redox-dependent injury in clinically defined uveitic entities. Clinical translation will also require validated biomarkers and evidence of ocular target engagement. Until such evidence is available, redox-directed therapies should be regarded as investigational adjuncts rather than established stand-alone treatments for uveitis.

## Figures and Tables

**Table 1:** Major reactive species, oxidative molecular damage markers, and composite redox measures relevant to uveitis research.

Reactive Species or Marker	Classification	Major Sources Relevant to Uveitis	Principal Molecular Targets or Biological Meaning	Assessment Approaches	Major Limitations	Key References
Superoxide anion (O_2_^•−^)	Reactive oxygen species	NOX2/Ncf1-associated oxidative responses in infiltrating phagocytes; mitochondrial electron leakage in retinal cells; activated neutrophils	Precursor of H_2_O_2_ and other secondary oxidants; reacts with nitric oxide; contributes to redox-sensitive inflammatory signaling and mitochondrial injury	Dihydroethidium, MitoSOX, chemiluminescence, or electron paramagnetic resonance (EPR)	Fluorescent probes have limited species specificity, may undergo nonspecific oxidation, and do not reliably identify the cellular or enzymatic source of O_2_^•−^ in vivo.	[14,42]
Hydrogen peroxide (H_2_O_2_)	Reactive oxygen species	Superoxide dismutation and mitochondrial- or NOX-associated redox reactions	Oxidation of redox-sensitive cysteine residues; modulation of NF-κB and other signaling pathways; contribution to macromolecular damage at high concentrations	Amplex Red-based assays or genetically encoded H_2_O_2_ probes	H_2_O_2_ is diffusible and rapidly metabolized, and its concentration is highly compartment-dependent. Direct species-specific measurements in uveitis models or patient ocular samples remain limited.	[14,47] (indirect evidence through ROS pathway modulation)
Hydroxyl radical (^•^OH)	Highly reactive oxygen species	Metal-catalyzed reactions involving H_2_O_2_, including Fenton-type chemistry	Rapid and nonspecific oxidation of DNA, membrane lipids, and proteins	Electron paramagnetic resonance spin trapping	Direct measurement is difficult because ^•^OH has an extremely short half-life and reacts near its site of generation. In uveitis studies, its involvement is generally inferred from downstream oxidative damage products.	[42,48]
Nitric oxide-derived reactive species, including peroxynitrite (ONOO^−^)	Reactive nitrogen species	Reaction of O_2_^•−^ with nitric oxide; increased inducible nitric oxide synthase activity in inflamed retinal tissue and photoreceptor mitochondria	Protein tyrosine nitration, mitochondrial protein dysfunction, and nuclear or mitochondrial DNA damage	Nitrotyrosine immunostaining or assays of related nitration products	Nitrotyrosine is not exclusively generated by ONOO^−^ and does not identify the producing cell, temporal sequence, or precise reactive nitrogen species.	[15,42]
Malondialdehyde and related lipid peroxidation products	Oxidative molecular damage markers	Oxidation of polyunsaturated fatty acids in cellular and mitochondrial membranes	Reflect lipid peroxidation and membrane oxidative injury; reactive aldehydes may also form adducts with proteins and nucleic acids	Thiobarbituric acid-reactive substances assays, ELISA, HPLC, or mass spectrometry	Thiobarbituric acid-based methods have limited specificity. Serum measurements cannot establish that lipid peroxidation originated within the eye.	[49]
8-Hydroxy-2′-deoxyguanosine and mitochondrial DNA damage	Oxidative molecular damage markers	Oxidative or nitrosative injury to nuclear and mitochondrial DNA, particularly during mitochondrial dysfunction	Reflect oxidative modification of DNA and potential impairment of mitochondrial function	Immunostaining, ELISA, chromatography, mass spectrometry, or assays of mitochondrial DNA integrity	These measurements do not identify the responsible reactive species and may be influenced by DNA repair, sample handling, and cellular turnover.	[42]
Protein nitration	Oxidative/nitrosative molecular damage marker	Nitric oxide-derived oxidants generated during retinal inflammation and mitochondrial stress	Modification of tyrosine residues and possible impairment of mitochondrial and cellular protein function	Nitrotyrosine immunostaining or immunoblotting	Protein nitration is a relatively stable footprint of nitrosative stress but is not specific for a single reactive species or enzymatic source.	[15,42]
Total oxidant capacity and oxidative stress index	Composite redox measures	Combined contribution of multiple circulating or tissue oxidants	Provide an overall estimate of oxidant burden or the balance between total oxidant and antioxidant measures	Colorimetric assays	These indices do not identify individual reactive species, cellular sources, molecular targets, or specific oxidative molecular damage. Serum values may reflect systemic rather than intraocular processes.	[49]
Total antioxidant capacity	Composite antioxidant measure	Combined enzymatic and non-enzymatic antioxidant activity in serum or ocular fluids	Reflects overall antioxidant capacity and may represent antioxidant sufficiency or a compensatory response to inflammation	Composite colorimetric or related antioxidant-capacity assays	These assays cannot identify the responsible antioxidant molecule or determine whether increased capacity is protective, compensatory, or functionally sufficient. Results are affected by sample type and control-group selection.	[49,50]

Abbreviations: EPR, electron paramagnetic resonance; HPLC, high-performance liquid chromatography; NOX2, NADPH oxidase 2. Oxidative damage products and composite redox indices should not be interpreted as direct measurements of a specific reactive species or its cellular source.

**Table 2:** Oxidative stress-related pathways and cellular mechanisms implicated in uveitis.

Pathway	Main Cell Type(s)	Mechanism	Evidence Source/Model	Evidence in Uveitis	Potential Therapeutic Relevance	Key References
NF-κB signaling	Retinal cells; retinal endothelial cells; infiltrating immune cells	Links ROS to cytokine, chemokine, and adhesion molecule expression.	Human peripheral blood from patients with uveitis; mouse and rat EAU; mouse and rat EIU	ROS suppression, antioxidant treatment, or direct NF-κB inhibition reduced retinal NF-κB activation, inflammatory mediator expression, and disease severity in EAU and EIU. A study combining peripheral blood samples from patients with uveitis and rat EAU further implicated an oxidative stress–mtDNA–cGAS–STING–NF-κB axis.	Reduction in inflammatory amplification, leukocyte recruitment, and BRB dysfunction	[14,47,48,53,54,55,66]
Nrf2/HO-1 pathway	RPE cells; retinal cells; inflammatory cells	Induces antioxidant and cytoprotective responses and counter-regulates ROS-driven inflammation.	Mouse EAU; in vitro RPE-cell oxidative stress models	Nrf2 deficiency worsens EAU, whereas Nrf2 activation and Keap1/Nrf2/HO-1 modulation reduce ocular inflammation and oxidative stress.	Enhancement of endogenous antioxidant defense during uveitic inflammation	[72,73,74]
MAPK signaling	Retinal cells; RPE cells; CD4^+^ T cells	Translates oxidative and inflammatory stress into apoptosis, cytokine production, and uveitogenic T-cell responses.	Mouse EAU; human VKH patient-derived cells; general cellular and non-uveitic mechanistic studies	MAPK activation has been demonstrated in mouse EAU, and MAPK-directed interventions have modulated uveitogenic T-cell responses and disease severity in selected experimental studies. In PBMCs from patients with VKH disease and lymphocytes from EAU mice, p38α MAPK inhibition suppressed IL-17 production in vitro, although intraocular p38 inhibition did not prevent EAU in that study.	Attenuation of Th1/Th17 responses and retinal inflammatory injury	[58,59,60]
PI3K-Akt signaling	Retinal cells; CD4^+^ T cells	Regulates survival, metabolism, CD4^+^ T-cell glycolysis, Th17/Treg balance, and GM-CSF production.	Mouse EAU; ex vivo murine CD4^+^ T cells; general signaling studies	PI3K-Akt-related metabolic regulation contributes to uveitogenic T-cell responses and EAU severity.	Restraint of pathogenic immune–metabolic remodeling in autoimmune uveitis	[61]
ROS/TXNIP/HIF-1α axis	CD4^+^ T cells	Promotes Th17 differentiation and inflammatory activity while limiting Treg responses.	Mouse EAU; ex vivo murine CD4^+^ T cells	Melatonin reduces ROS accumulation, suppresses TXNIP/HIF-1α signaling, restores Th17/Treg balance, and attenuates EAU.	Restoration of Th17/Treg balance	[62]
NOX2/Ncf1-associated ROS response	Retinal myeloid cells; infiltrating phagocytes	Contributes to retinal oxidative injury, inflammatory mediator production, and NF-κB activation.	Mouse EAU; genetically modified Ncf1-deficient mice	Ncf1 deficiency or N-acetylcysteine treatment suppresses ROS responses and reduces EAU severity.	Reduction in myeloid-cell-associated inflammatory amplification	[14]
P2X7/NLRP3 inflammasome-related signaling	Mononuclear phagocytes; macrophages; microglia	Links ATP-mediated danger sensing to inflammasome activation, IL-1β/IL-18 release, ROS production, and Th17-related inflammation.	Mouse EAU with cell-specific P2X7 deletion; ex vivo murine macrophages and microglia; supporting immune-cell studies	P2X7 deficiency in mononuclear phagocytes reduces EAU severity, retinal immune-cell infiltration, IL-1β release, and pathogenic Th17 responses.	Suppression of macrophage/microglia-driven autoimmune retinal inflammation	[76,77,78]
ROS–VEGF–BRB axis	Retinal endothelial cells; RPE cells	Connects ROS, VEGF-related permeability, tight junction alteration, and leukocyte trafficking.	Mouse EAU for BRB dysfunction; in vitro retinal endothelial and RPE-cell models; indirect evidence from DR and other retinal vascular diseases	Direct evidence for BRB dysfunction and leukocyte trafficking exists in EAU; the specific ROS–VEGF–BRB relationship is inferred mainly from retinal vascular and in vitro barrier studies and has not been directly established in uveitis.	Hypothesis-generating target for preservation of BRB integrity; direct validation in uveitis is required.	[43,44,79,80,82,83,84]

**Table 3:** Human evidence for redox abnormalities and redox-sensitive pathways across uveitic entities.

Uveitic Entity	Human Biological Sample Assessed	Redox-Related Marker(s) or Pathway Assessed	Main Observation	Major Limitation	Interpretation	Key References
Behçet’s uveitis	Circulating neutrophils isolated from peripheral blood	Neutrophil phenotype and oxidative-burst-related biology	Disease-specific neutrophil abnormalities have been identified in patients with Behçet’s uveitis, while broader Behçet’s syndrome literature supports a relationship between neutrophil oxidative responses and vascular inflammation.	ROS were not measured in aqueous humor, vitreous, retinal vessels, or ocular tissue; part of the oxidative-burst evidence derives from systemic Behçet’s syndrome rather than ocular disease specifically.	Disease-specific human evidence supports biological plausibility, but direct intraocular redox involvement remains unproven.	[45,46]
Acute anterior uveitis	Serum	Total oxidant capacity, oxidative stress index, malondialdehyde, and total antioxidant capacity	Serum total oxidant capacity, oxidative stress index, and malondialdehyde were increased during a first episode of acute anterior uveitis, whereas total antioxidant capacity did not differ significantly from controls.	The cohort was etiologically heterogeneous, measurements were systemic rather than intraocular, and patients were not stratified according to HLA-B27 status.	Supports an association between active acute anterior uveitis and systemic redox imbalance and lipid peroxidation.	[49]
HLA-B27-associated acute anterior uveitis	No disease-specific human sample assessed	No disease-specific redox marker assessed	No study identified in this review directly compared oxidative markers between HLA-B27-positive and HLA-B27-negative acute anterior uveitis.	The available acute anterior uveitis cohort was not stratified by HLA-B27 status; clinical characteristics of HLA-B27-associated disease do not themselves establish redox involvement.	A disease-specific role for oxidative stress remains unproven.	[9,49] *
Vogt–Koyanagi–Harada disease	Patient-derived peripheral blood immune cells	p38α MAPK–IL-17 signaling	p38α MAPK was shown to regulate IL-17 production in cells obtained from patients with VKH disease and in EAU.	ROS production and oxidative molecular damage were not directly measured; p38 MAPK can be activated by multiple inflammatory and stress-related stimuli.	Supports involvement of a potentially redox-sensitive inflammatory pathway, but does not prove disease-specific oxidative stress.	[60]
Mixed infectious and non-infectious uveitis cohort	Aqueous humor	PEDF and total antioxidant capacity	Aqueous humor PEDF was positively correlated with total antioxidant capacity, and both were higher in uveitis than in proliferative diabetic retinopathy.	No healthy ocular control group was included; infectious and non-infectious uveitis were pooled; total antioxidant capacity does not identify a specific reactive species, cellular source, or oxidative damage product.	May reflect compensatory intraocular antioxidant regulation rather than a uniform antioxidant deficiency.	[50]
Clinically heterogeneous uveitis cohort	Peripheral blood	ROS, mitochondrial dysfunction, cytosolic mtDNA, and cGAS–STING–NF-κB signaling	Increased ROS, mitochondrial dysfunction, cytosolic mtDNA accumulation, and pathway activation were reported in peripheral blood samples from patients with uveitis.	The human cohort was not sufficiently characterized by specific uveitic entity; mechanistic causality and therapeutic effects were established mainly in the rat EAU component of the study.	Provides preliminary human evidence of systemic redox and mitochondrial abnormalities, but disease-specific and intraocular relevance remains uncertain.	[47]

* References [9,49] provide clinical and cohort context only; neither establishes an HLA-B27-specific redox mechanism.

## Data Availability

No new data were created or analyzed in this study. Data sharing is not applicable to this article.

## References

[1] Burkholder B.M., Jabs D.A. (2021). Uveitis for the non-ophthalmologist. BMJ.

[2] Jabs D.A., Busingye J. (2013). Approach to the diagnosis of the uveitides. Am. J. Ophthalmol..

[3] Tomkins-Netzer O., Niederer R., Greenwood J., Fabian I.D., Serlin Y., Friedman A., Lightman S. (2024). Mechanisms of blood-retinal barrier disruption related to intraocular inflammation and malignancy. Prog. Retin. Eye Res..

[4] Ferreira L.B., Williams K.A., Best G., Haydinger C.D., Smith J.R. (2023). Inflammatory cytokines as mediators of retinal endothelial barrier dysfunction in non-infectious uveitis. Clin. Transl. Immunol..

[5] Kim J., Chun J., Ahn M., Jung K., Moon C., Shin T. (2022). Blood-retina barrier dysfunction in experimental autoimmune uveitis: The pathogenesis and therapeutic targets. Anat. Cell Biol..

[6] Maghsoudlou P., Epps S.J., Guly C.M., Dick A.D. (2025). Uveitis in Adults: A Review. JAMA.

[7] Zong Y., Tong X., Chong W.P. (2025). Th17 Response in Uveitis: A Double-Edged Sword in Ocular Inflammation and Immune Regulation. Clin. Rev. Allergy Immunol..

[8] Zhang M., Zhang X. (2023). T cells in ocular autoimmune uveitis: Pathways and therapeutic approaches. Int. Immunopharmacol..

[9] Chang J.H., McCluskey P.J., Wakefield D. (2005). Acute anterior uveitis and HLA-B27. Surv. Ophthalmol..

[10] Sies H., Berndt C., Jones D.P. (2017). Oxidative Stress. Annu. Rev. Biochem..

[11] Bohm E.W., Buonfiglio F., Voigt A.M., Bachmann P., Safi T., Pfeiffer N., Gericke A. (2023). Oxidative stress in the eye and its role in the pathophysiology of ocular diseases. Redox Biol..

[12] Chistyakov D.V., Tiulina V.V., Gancharova O.S., Baksheeva V.E., Goriainov S.V., Shebardina N.G., Ivlev V.A., Komarov S.V., Shevelyova M.P., Tikhomirova N.K. (2024). Targeting Oxidative Stress and Inflammation in the Eye: Insights from a New Model of Experimental Autoimmune Uveitis. Int. J. Mol. Sci..

[13] Ung L., Pattamatta U., Carnt N., Wilkinson-Berka J.L., Liew G., White A.J.R. (2017). Oxidative stress and reactive oxygen species: A review of their role in ocular disease. Clin. Sci..

[14] Hsu S.M., Yang C.H., Teng Y.T., Tsai H.Y., Lin C.Y., Lin C.J., Shieh C.C., Chen S.H. (2020). Suppression of the Reactive Oxygen Response Alleviates Experimental Autoimmune Uveitis in Mice. Int. J. Mol. Sci..

[15] Nguyen A.M., Rao N.A. (2011). Oxidative photoreceptor cell damage in autoimmune uveitis. J. Ophthalmic Inflamm. Infect..

[16] Poprac P., Jomova K., Simunkova M., Kollar V., Rhodes C.J., Valko M. (2017). Targeting Free Radicals in Oxidative Stress-Related Human Diseases. Trends Pharmacol. Sci..

[17] Prasad S., Gupta S.C., Tyagi A.K. (2017). Reactive oxygen species (ROS) and cancer: Role of antioxidative nutraceuticals. Cancer Lett..

[18] Sabharwal S.S., Schumacker P.T. (2014). Mitochondrial ROS in cancer: Initiators, amplifiers or an Achilles’ heel?. Nat. Rev. Cancer.

[19] Halliwell B. (2011). Free radicals and antioxidants—Quo vadis?. Trends Pharmacol. Sci..

[20] Pham-Huy L.A., He H., Pham-Huy C. (2008). Free radicals, antioxidants in disease and health. Int. J. Biomed. Sci..

[21] Chen Y., Mehta G., Vasiliou V. (2009). Antioxidant defenses in the ocular surface. Ocul. Surf..

[22] Saccà S.C., Roszkowska A.M., Izzotti A. (2013). Environmental light and endogenous antioxidants as the main determinants of non-cancer ocular diseases. Mutat. Res. Mutat. Res..

[23] Saccà S.C., Cutolo C.A., Ferrari D., Corazza P., Traverso C.E. (2018). The eye, oxidative damage and polyunsaturated fatty acids. Nutrients.

[24] Catala A. (2011). Lipid peroxidation of membrane phospholipids in the vertebrate retina. Front. Biosci..

[25] Kang Q., Yang C. (2020). Oxidative stress and diabetic retinopathy: Molecular mechanisms, pathogenetic role and therapeutic implications. Redox Biol..

[26] Ozawa Y. (2020). Oxidative stress in the light-exposed retina and its implication in age-related macular degeneration. Redox Biol..

[27] Dan Dunn J., Alvarez L.A., Zhang X., Soldati T. (2015). Reactive oxygen species and mitochondria: A nexus of cellular homeostasis. Redox Biol..

[28] Letts J.A., Sazanov L.A. (2017). Clarifying the supercomplex: The higher-order organization of the mitochondrial electron transport chain. Nat. Struct. Mol. Biol..

[29] Letts J.A., Fiedorczuk K., Sazanov L.A. (2016). The architecture of respiratory supercomplexes. Nature.

[30] Forbes J.M., Thorburn D.R. (2018). Mitochondrial dysfunction in diabetic kidney disease. Nat. Rev. Nephrol..

[31] Brieger K., Schiavone S., Miller F.J., Krause K.H. (2012). Reactive oxygen species: From health to disease. Swiss Med. Wkly..

[32] Manea S.A., Constantin A., Manda G., Sasson S., Manea A. (2015). Regulation of Nox enzymes expression in vascular pathophysiology: Focusing on transcription factors and epigenetic mechanisms. Redox Biol..

[33] Förstermann U., Xia N., Li H. (2017). Roles of Vascular Oxidative Stress and Nitric Oxide in the Pathogenesis of Atherosclerosis. Circ. Res..

[34] Dammak A., Pastrana C., Martin-Gil A., Carpena-Torres C., Peral Cerda A., Simovart M., Alarma P., Huete-Toral F., Carracedo G. (2023). Oxidative Stress in the Anterior Ocular Diseases: Diagnostic and Treatment. Biomedicines.

[35] Kuntic M., Kuntic I., Krishnankutty R., Gericke A., Oelze M., Junglas T., Bayo Jimenez M.T., Stamm P., Nandudu M., Hahad O. (2023). Co-exposure to urban particulate matter and aircraft noise adversely impacts the cerebro-pulmonary-cardiovascular axis in mice. Redox Biol..

[36] Hahad O., Kuntic M., Kuntic I., Daiber A., Münzel T. (2023). Tobacco smoking and vascular biology and function: Evidence from human studies. Pflug. Arch..

[37] Guo C., Ning X., Zhang J., Zhang C., Wang J., Su L., Han J., Ma N. (2023). Ultraviolet B radiation induces oxidative stress and apoptosis in human lens epithelium cells by activating NF-κB signaling to down-regulate sodium vitamin C transporter 2 (SVCT2) expression. Cell Cycle.

[38] Bayo Jimenez M.T., Gericke A., Frenis K., Rajlic S., Kvandova M., Kröller-Schön S., Oelze M., Kuntic M., Kuntic I., Mihalikova D. (2023). Effects of aircraft noise cessation on blood pressure, cardio- and cerebrovascular endothelial function, oxidative stress, and inflammation in an experimental animal model. Sci. Total Environ..

[39] Barbier E., Carpentier J., Simonin O., Gosset P., Platel A., Happillon M., Alleman L.Y., Perdrix E., Riffault V., Chassat T. (2023). Oxidative stress and inflammation induced by air pollution-derived PM(2.5) persist in the lungs of mice after cessation of their sub-chronic exposure. Environ. Int..

[40] de Jager T.L., Cockrell A.E., Du Plessis S.S. (2017). Ultraviolet Light Induced Generation of Reactive Oxygen Species. Adv. Exp. Med. Biol..

[41] Marchitti S.A., Chen Y., Thompson D.C., Vasiliou V. (2011). Ultraviolet radiation: Cellular antioxidant response and the role of ocular aldehyde dehydrogenase enzymes. Eye Contact Lens.

[42] Rajendram R., Saraswathy S., Rao N.A. (2007). Photoreceptor mitochondrial oxidative stress in early experimental autoimmune uveoretinitis. Br. J. Ophthalmol..

[43] Quinn J., Salman A., Paluch C., Jackson-Wood M., McClements M.E., Luo J., Davis S.J., Cornall R.J., MacLaren R.E., Dendrou C.A. (2024). Single-cell transcriptomic analysis of retinal immune regulation and blood-retinal barrier function during experimental autoimmune uveitis. Sci. Rep..

[44] Xu H., Forrester J.V., Liversidge J., Crane I.J. (2003). Leukocyte trafficking in experimental autoimmune uveitis: Breakdown of blood-retinal barrier and upregulation of cellular adhesion molecules. Investig. Ophthalmol. Vis. Sci..

[45] Liu R., Wang Q., Jiang Q., Chang R., Zhou Y., Ye X., Luo X., Lai Y., Su G., Yang P. (2025). Proteomic Profiles of Neutrophils from Behcet’s Uveitis Patients and their Sex Differences. Inflammation.

[46] Emmi G., Becatti M., Bettiol A., Hatemi G., Prisco D., Fiorillo C. (2019). Behcet’s Syndrome as a Model of Thrombo-Inflammation: The Role of Neutrophils. Front. Immunol..

[47] Xu S., Feng J., Peng Y., Xu F., Liu Y., Xie Y., Liu B., Yin X., Wei H., Tian Q. (2026). Tempol alleviates autoimmune uveitis by targeting the oxidative stress-mediated cGAS-STING-NF-kB signaling pathway to restore M1/M2 macrophage polarization balance. Int. Immunopharmacol..

[48] Kubota S., Kurihara T., Mochimaru H., Satofuka S., Noda K., Ozawa Y., Oike Y., Ishida S., Tsubota K. (2009). Prevention of ocular inflammation in endotoxin-induced uveitis with resveratrol by inhibiting oxidative damage and nuclear factor-κB activation. Investig. Ophthalmol. Vis. Sci..

[49] Turk A., Aykut M., Akyol N., Kola M., Mentese A., Sumer A., Alver A., Erdol H. (2014). Serum anti-carbonic anhydrase antibodies and oxidant-antioxidant balance in patients with acute anterior uveitis. Ocul. Immunol. Inflamm..

[50] Yoshida Y., Yamagishi S., Matsui T., Nakamura K., Imaizumi T., Yoshimura K., Yamakawa R. (2007). Positive correlation of pigment epithelium-derived factor and total antioxidant capacity in aqueous humour of patients with uveitis and proliferative diabetic retinopathy. Br. J. Ophthalmol..

[51] Naik E., Dixit V.M. (2011). Mitochondrial reactive oxygen species drive proinflammatory cytokine production. J. Exp. Med..

[52] Jassim A.H., Inman D.M., Mitchell C.H. (2021). Crosstalk between dysfunctional mitochondria and inflammation in glaucomatous neurodegeneration. Front. Pharmacol..

[53] Kitamei H., Iwabuchi K., Namba K., Yoshida K., Yanagawa Y., Kitaichi N., Kitamura M., Ohno S., Onoé K. (2006). Amelioration of experimental autoimmune uveoretinitis (EAU) with an inhibitor of nuclear factor-κB (NF-κB), pyrrolidine dithiocarbamate. J. Leukoc. Biol..

[54] Iwata D., Kitaichi N., Miyazaki A., Iwabuchi K., Yoshida K., Namba K., Ozaki M., Ohno S., Umezawa K., Yamashita K. (2010). Amelioration of experimental autoimmune uveoretinitis with nuclear factor-κB Inhibitor dehydroxy methyl epoxyquinomicin in mice. Investig. Ophthalmol. Vis. Sci..

[55] Hsu S.M., Yang C.H., Tsai H.Y., Lin C.J., Fang Y.H., Shieh C.C., Chen S.H. (2020). Chitosan Oligosaccharides Suppress Nuclear Factor-Kappa B Activation and Ameliorate Experimental Autoimmune Uveoretinitis in Mice. Int. J. Mol. Sci..

[56] Brazil D.P., Yang Z.-Z., Hemmings B.A. (2004). Advances in protein kinase B signalling: AKTion on multiple fronts. Trends Biochem. Sci..

[57] Semenza G.L. (2012). Hypoxia-inducible factors in physiology and medicine. Cell.

[58] Qiu Y., Tao L., Zheng S., Lin R., Fu X., Chen Z., Lei C., Wang J., Li H., Li Q. (2016). AAV8-Mediated Angiotensin-Converting Enzyme 2 Gene Delivery Prevents Experimental Autoimmune Uveitis by Regulating MAPK, NF-κB and STAT3 Pathways. Sci. Rep..

[59] Hu Y., Chen G., Huang J., Li Z., Li Z., Xie Y., Chen Y., Li H., Su W., Chen X. (2021). The Calcium Channel Inhibitor Nimodipine Shapes the Uveitogenic T Cells and Protects Mice from Experimental Autoimmune Uveitis through the p38-MAPK Signaling Pathway. J. Immunol..

[60] Commodaro A.G., Bombardieri C.R., Peron J.P., Saito K.C., Guedes P.M., Hamassaki D.E., Belfort R.N., Rizzo L.V., Belfort R., de Camargo M.M. (2010). p38α MAP kinase controls IL-17 synthesis in vogt-koyanagi-harada syndrome and experimental autoimmune uveitis. Investig. Ophthalmol. Vis. Sci..

[61] Li Z., Duan R., Jiang Q., Liu J., Chen J., Jiang L., Wang T., Li H., Zhang Y., Peng X. (2024). Dietary caloric restriction protects experimental autoimmune uveitis by regulating Teff/Treg balance. iScience.

[62] Huang J., Li Z., Hu Y., Li Z., Xie Y., Huang H., Chen Q., Chen G., Zhu W., Chen Y. (2022). Melatonin, an endogenous hormone, modulates Th17 cells via the reactive-oxygen species/TXNIP/HIF-1α axis to alleviate autoimmune uveitis. J. Neuroinflammation.

[63] Cai Z.-Y., Fu M.-D., Liu K., Duan X.-C. (2021). Therapeutic effect of Keap1-Nrf2-ARE pathway-related drugs on age-related eye diseases through anti-oxidative stress. Int. J. Ophthalmol..

[64] Napetschnig J., Wu H. (2013). Molecular basis of NF-κB signaling. Annu. Rev. Biophys..

[65] Morgan M.J., Liu Z.G. (2011). Crosstalk of reactive oxygen species and NF-κB signaling. Cell Res..

[66] Yadav U.C., Subramanyam S., Ramana K.V. (2009). Prevention of endotoxin-induced uveitis in rats by benfotiamine, a lipophilic analogue of vitamin B1. Investig. Ophthalmol. Vis. Sci..

[67] Zhong Q., Mishra M., Kowluru R.A. (2013). Transcription factor Nrf2-mediated antioxidant defense system in the development of diabetic retinopathy. Investig. Ophthalmol. Vis. Sci..

[68] Pae H.O., Chung H.T. (2009). Heme oxygenase-1: Its therapeutic roles in inflammatory diseases. Immune Netw..

[69] Ryter S.W. (2020). Therapeutic Potential of Heme Oxygenase-1 and Carbon Monoxide in Acute Organ Injury, Critical Illness, and Inflammatory Disorders. Antioxidants.

[70] Naito Y., Takagi T., Uchiyama K., Yoshikawa T. (2011). Heme oxygenase-1: A novel therapeutic target for gastrointestinal diseases. J. Clin. Biochem. Nutr..

[71] Ryter S.W., Choi A.M. (2009). Heme oxygenase-1/carbon monoxide: From metabolism to molecular therapy. Am. J. Respir. Cell Mol. Biol..

[72] Woo J.M., Shin D.Y., Lee S.J., Joe Y., Zheng M., Yim J.H., Callaway Z., Chung H.T. (2012). Curcumin protects retinal pigment epithelial cells against oxidative stress via induction of heme oxygenase-1 expression and reduction of reactive oxygen. Mol. Vis..

[73] Tang W., Huang X., Yi Y.D., Cao F., Deng M., Fan J., Jiang Z.X., Tao L.M., Wang X., Shi L. (2025). Hyaluronic acid-curcumin nanoparticles for preventing the progression of experimental autoimmune uveitis through the Keap1/Nrf2/HO-1 signaling pathway. J. Nanobiotechnol..

[74] Sato Y., Saito S., Nakayama M., Sugita S., Kudo A., Keino H. (2022). Genetic Ablation of Nrf2 Exacerbates Neuroinflammation in Ocular Autoimmunity. Int. J. Mol. Sci..

[75] Kyriakis J.M., Avruch J. (2001). Mammalian mitogen-activated protein kinase signal transduction pathways activated by stress and inflammation. Physiol. Rev..

[76] Déchelle-Marquet P.A., Che Y., Roux C., Blond F., Ronning K.E., Augustin S., Lagouge-Roussey P., Nous C., Touhami S., Bodaghi B. (2025). Macrophage expression of P2X7 controls autoimmune uveitis. J. Neuroinflamm..

[77] Di Virgilio F., Dal Ben D., Sarti A.C., Giuliani A.L., Falzoni S. (2017). The P2X7 Receptor in Infection and Inflammation. Immunity.

[78] Ferrari D., Chiozzi P., Falzoni S., Dal Susino M., Melchiorri L., Baricordi O.R., Di Virgilio F. (1997). Extracellular ATP triggers IL-1 beta release by activating the purinergic P2Z receptor of human macrophages. J. Immunol..

[79] Cunha-Vaz J., Bernardes R., Lobo C. (2011). Blood-retinal barrier. Eur. J. Ophthalmol..

[80] O’Leary F., Campbell M. (2023). The blood-retina barrier in health and disease. FEBS J..

[81] Jin X.H., Ohgami K., Shiratori K., Suzuki Y., Hirano T., Koyama Y., Yoshida K., Ilieva I., Iseki K., Ohno S. (2006). Inhibitory effects of lutein on endotoxin-induced uveitis in Lewis rats. Investig. Ophthalmol. Vis. Sci..

[82] Xu H., Dawson R., Crane I.J., Liversidge J. (2005). Leukocyte diapedesis in vivo induces transient loss of tight junction protein at the blood-retina barrier. Investig. Ophthalmol. Vis. Sci..

[83] Bailey T.A., Kanuga N., Romero I.A., Greenwood J., Luthert P.J., Cheetham M.E. (2004). Oxidative stress affects the junctional integrity of retinal pigment epithelial cells. Investig. Ophthalmol. Vis. Sci..

[84] Deissler H.L., Deissler H., Lang G.K., Lang G.E. (2013). VEGF but not PlGF disturbs the barrier of retinal endothelial cells. Exp. Eye Res..

[85] Suzuki Y., Ohgami K., Shiratori K., Jin X.H., Ilieva I., Koyama Y., Yazawa K., Yoshida K., Kase S., Ohno S. (2006). Suppressive effects of astaxanthin against rat endotoxin-induced uveitis by inhibiting the NF-κB signaling pathway. Exp. Eye Res..

[86] Van Rooij J., Schwartzenberg S.G., Mulder P.G., Baarsma S.G. (1999). Oral vitamins C and E as additional treatment in patients with acute anterior uveitis: A randomised double masked study in 145 patients. Br. J. Ophthalmol..

